# The Effect of Unerupted Permanent Tooth Crowns on the Distribution of Masticatory Stress in Children

**DOI:** 10.1371/journal.pone.0029121

**Published:** 2011-12-14

**Authors:** Ashley S. Hammond, Elizabeth R. Dumont, Robert C. McCarthy

**Affiliations:** 1 Department of Pathology and Anatomical Sciences, University of Missouri School of Medicine, Columbia, Missouri, United States of America; 2 Department of Anthropology, Florida Atlantic University, Boca Raton, Florida, United States of America; 3 Department of Organismal and Evolutionary Biology, University of Massachusetts-Amherst, Amherst, Massachusetts, United States of America; 4 Department of Anthropology, University at Albany, Albany, New York, United States of America; University of Utah, United States of America

## Abstract

Human mothers wean their children from breast milk at an earlier developmental stage than do ape mothers, resulting in human children chewing solid and semi-solid foods using the deciduous dentition. Mechanical forces generated by chewing solid foods during the post-weaning period travel through not only the deciduous teeth, but also the enamel caps of the developing permanent teeth within the maxilla and mandible, which are not present in the adult face. The effects of mechanical stress propagating through these very stiff structures have yet to be examined. Based on a heuristic model, we predicted that the enamel of the embedded developing teeth would act to reduce stresses in the surrounding bony elements of the juvenile face. We tested this hypothesis by simulating occlusal loading in a finite element (FE) model of a child's cranium with a complete set of deciduous teeth and the first permanent molars embedded in the bony crypt in the maxilla. We modeled bone and enamel with appropriate material properties and assessed the effect of embedding high-stiffness enamel structures on stress distribution in the juvenile face. Against expectation, the presence of unerupted enamel caps does not affect the magnitude or location of stresses in the juvenile face. Our results do not support the hypothesis that the unerupted secondary teeth act to moderate stresses in the juvenile face.

## Introduction

Weaning, or the transition from breast milk to the habitual consumption of adult foods [1], occurs at an earlier developmental stage in humans than in other primates. Chimpanzees and other great apes wean between three and seven years of age [2], [3], which is coincident with eruption of the first permanent molar, the asymptotic conclusion of brain growth, and maturation of the digestive and masticatory systems [4]–[7]. Modern humans wean earlier, ranging from about one year in industrialized countries to four years in some natural fertility populations [2], [3], [8]. Species-typical weaning in modern humans therefore occurs two to seven years prior to eruption of the permanent dentition [9] and numerous other markers of biological and digestive maturity that occur between six and eight years of age [4], [5], [10], [11]. This means that human children primarily use their deciduous, not their permanent, dentition to masticate post-weaning childhood foods.

The overall configuration of the adult human skull allows it to generate high bite forces during chewing and to effectively dissipate masticatory forces [12]–[17]. However, unlike adults, the facial skeleton of juveniles contains unerupted permanent teeth that could affect the distribution of stress in the face during feeding. The developing tooth caps are in approximately the same areas as bony trabecular struts in the adult maxilla and mandible [18]–[20]. Furthermore, the unerupted secondary teeth are located in tooth crypts that are in very close proximity to or touching the alveolar bone, particularly as the teeth approach adult dimensions. As teeth begin the eruption process, the bony crypts begin to fill with bone [21], further reducing the space around the developing teeth in the maxilla and mandible. Although the elastic modulus and Poisson's ratio of developing enamel and the tooth crypt itself are unknown, the enamel caps present during the first years of life are known to be substantially more mineralized than the surrounding bone [22]. Moreover, a recent study of a macaque mandible demonstrated that the presence or absence of air-filled crypts affects levels of strain within the mandibular symphysis [23]. One interesting functional hypothesis is that the permanent tooth caps embedded in the juvenile face act to moderate stress in the craniofacial region during mastication, allowing modern human children to process adult foods with their deciduous teeth at an earlier developmental stage than do apes.

**Figure 1:**
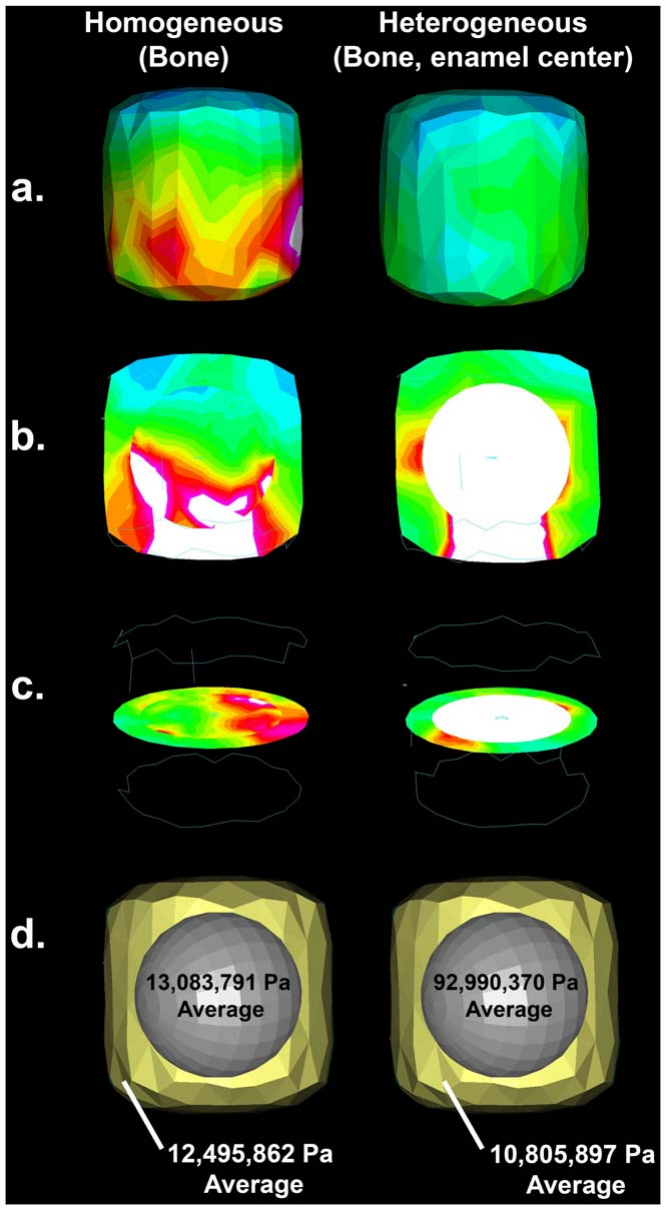
Geometric abstraction. Finite element analyses demonstrating the effect of a stiff object (the sphere) embedded within a less-stiff object on von Mises stress during loading. Side-views (a) and cross-sections (b,c) are presented for a model with a stiff center (heterogeneous) and an identical model with the material properties of cortical bone throughout (homogeneous). The solved models indicate a reduction in average von Mises stress in the outer bony shell in the homogeneous model, with increased von Mises stress found in the stiffer center relative to the homogenous model (d). Results are scaled from 0 to 2.0×10^7^ Pa, with highest stresses indicated by white regions.

Comparative biologists have only recently begun to explore how stress propagates through heterogeneous biological structures [17], [24]–[26]. Enamel and bone differ in Poisson's ratio, a measure of a material's compressibility, and Young's modulus, a measure of elasticity. We predicted that the stiff enamel caps within the softer maxillary bone affect the distribution of stress in the juvenile face under masticatory loads. This prediction can be illustrated using a finite element analysis (FEA) of a simple geometric object. We assigned different material properties to the spherical centers of two identical models (Figure 1). In the homogeneous model (left), we assigned Young's modulus and Poisson's ratio values appropriate for bone to both the spherical center and surrounding block (9.10 GPa elastic modulus, 0.38 Poisson's ratio [27]). In the heterogeneous model (right), we assigned the same Young's modulus and Poisson's ratio to the surrounding block, and values appropriate for enamel to the spherical center (77.90 GPa elastic modulus, 0.33 Poisson's ratio [28]). We then applied one Newton (N) of vertical force at the precise center of the inferior surface of both models, and constrained a node on the superior surfaces of the models in all directions to prevent rigid body motion. Von Mises stress values are far greater in the spherical center of the heterogeneous model, while average stresses for all tetrahedrals in the surrounding block are substantially lower. In this example, the presence of a stiff structure at the center of a more elastic block decreased the stress within the surrounding material.

By extension of this simple model, we hypothesized that the presence of the developing permanent tooth caps serve to reduce stresses in the juvenile face during loading. To test this hypothesis, we assessed the pattern and magnitude of stress in two FE models of a juvenile human face differing only in material properties of the tooth crypts. We loaded the deciduous first incisor in one trial and the deciduous first molar in a second trial.

## Materials and Methods

We selected specimen 14 from the Bosma CT Collection (see [29]) for model creation. It was important for this study to select a specimen older than the species-typical age at weaning (>2.5 years of age) that possess (1) a complete set of deciduous teeth, (2) unerupted secondary teeth in an advanced stage of development, and (3) developing teeth that are large relative to the surrounding space in the tooth crypts. All unerupted teeth had well developed crowns with a high radio-opacity, indicating that they were highly mineralized. Based on dental development and eruption chronologies [30]–[33], Bosma specimen 14 can be estimated to be four years of age. Bosma specimen 14 appeared normal in all regards, with no evidence of visible pathology. The CT series for this skull is composed of 95 axial slices in 1.5 mm increments, with a 512*512 resolution and 0.3105 pixel size.

The modeling techniques used for generating our finite element model have been described fully elsewhere [34], [35]. Briefly, we used a series of three software tools to generate a finite element mesh from the CT scans. We used VGStudio MAX to segment the cranium and the developing permanent tooth caps, to segment and remove the mandible, and to generate surface models of the skull and tooth caps. We then imported the surface models into Geomagic Studio v. 9, a 3D shape-processing and reconstruction tool. Geomagic was used to fix minor segmentation errors and to smooth the model. We also deleted the nasal conchae to facilitate mesh generation, a common practice in testing finite element models of biological structures [34], [36], [37], as these thin bony elements are unlikely to be load-bearing. We then exported the surface model of the cranium and embedded enamel tooth caps as a binary stereolithography file to Strand7 (Release 2.3, Sydney, Australia), where we generated a solid mesh of 4-noded tetrahedral elements. The model was composed of 851,840 tetrahedrals, of which 68,933 tetrahedrals compose the segmented tooth caps.

We duplicated the model to yield two identical models. We then assigned different material properties to the two models using published values for primate maxillary cortical bone [27] and enamel [24], [28], [38] (Table 1). In both models, we assigned the visible deciduous tooth crowns the properties of enamel, and the remainder of the cranium the properties of maxillary cortical bone. In one model, hereafter called ‘heterogeneous’ model, we assigned the developing tooth caps the properties of enamel. In the second, ‘homogeneous’ model, we assigned the developing tooth caps the properties of cortical bone, thus producing a model where the maxilla and embedded tooth caps are modeled with the same material properties. To summarize, the homogeneous and heterogeneous models are identical in all respects (number of tetrahedrals, load magnitudes, points of loading, loading constraints) except the material properties of the unerupted secondary teeth. The terms homogeneous and heterogeneous used to describe these models only refer to this difference in the material properties of the unerupted teeth; neither of our models included complex, orthotropic material models. These models contain, at most, two isotropic material properties, and therefore do not model the full complexity of tissues in a developing skull, such as the soft tissues in the tooth crypts and alveoli. Following [24], we chose to model only two tissue properties in order to decrease the probability of error associated with modeling soft-tissue structures from a CT series obtained from a dry skull.

**Table 1:** Model material properties*.

		Bone		Secondary Teeth	
Model	Total Load (N)	Modulus (GPa)	Poisson's Ratio	Modulus (GPa)	Poisson's Ratio
Heterogeneous	350	9.1	0.38	77.9	0.33
Homogeneous	350	9.1	0.38	9.1	0.38

*Values following [27], [28].

There are little data on either bite force or masticatory muscle activity during chewing in children [39]–[42]. Therefore, we did not apply muscle forces to the FE models but instead fixed the models and applied forces to the teeth. We applied two loading regimes to each model. The first loaded the deciduous right upper central incisor (di^1^) and the second applied a load on the deciduous right upper first molar (dm^1^). For each trial we identified five nodes on either the di^1^ or dm^1^ and applied a load of 70 N in the Z-axis (perpendicular to the bony palate), for a 350-N total force per trial. We constrained the models against rigid body motion in x, y, and z axes at three nodes within each mandibular glenoid fossa, and derived a finite element solution using a linear static analysis.

**Figure 2:**
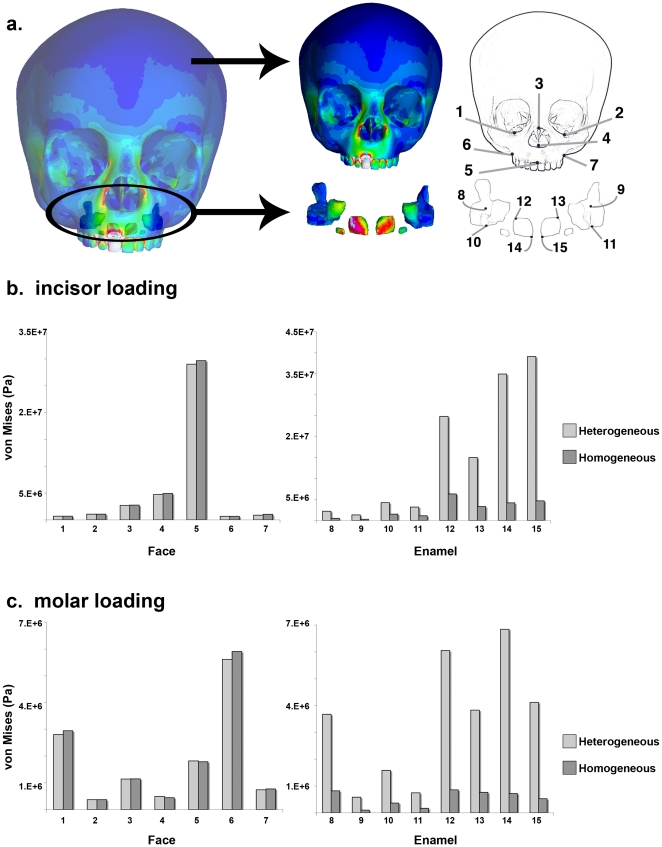
Comparison of von Mises stress by landmark. Von Mises stress values are shown for fifteen landmarks from the face and the embedded enamel caps (a). Heterogeneous and homogeneous models under the incisal loads (b) and molar loads (c) differ in von Mises stress calculated in the developing enamel caps, but not the face. Tooth cap images in (a) are not shown to scale.

The von Mises criterion for material failure, a calculation of maximum yield of materials under multiaxial loading via the principal stresses, is often used to express FE results when modeling elastic biological materials [34], [43]. In order to compare regional variation in stress between the two models quantitatively, we measured von Mises stresses at seven identical nodes on the craniofacial region and eight identical nodes on the secondary tooth caps (Table 2; Figure 2). As the results are presented in the same scale for all the models, the patterns and magnitudes of stress are comparable across models and loading regimes.

**Table 2:** Location of points sampled.

Landmark	Tetrahedral	Location
1	214,332	most inferior and anterior point on right orbital margin (orbitale)
2	45,755	most inferior and anterior point onleft orbital margin (orbitale)
3	10,461	midline point at inferior free end of internasal suture (rhinion)
4	633,483	midline point on inferior nasal aperature (nasospinale)
5	76,954	midline point at inferior bony septum between maxillary incisors (alveolare)
6	34,492	midpoint between base of the right zygomatic arch and the buccal sideof right M^2^
7	233,634	midpoint between base of the left zygomatic arch and the buccal sideof left M^2^
8	796,604	superior center of the right permanent M^2^ tooth cap
9	817,383	superior center of the left permanent M^2^ tooth cap
10	795,808	inferior (occlusal) center of the right permanent M^2^ tooth cap
11	812,817	inferior (occlusal) center of the left permanent M^2^ tooth cap
12	790,006	most superior point on right labial surface of right I^1^ tooth cap
13	851,886	most superior point on left labial surface of left I^1^ tooth cap
14	794,686	most inferior point on right labial surface of right I^1^ tooth cap
15	851,410	most inferior point on left labial surface of left I^1^ tooth cap

## Results

**Figure 3:**
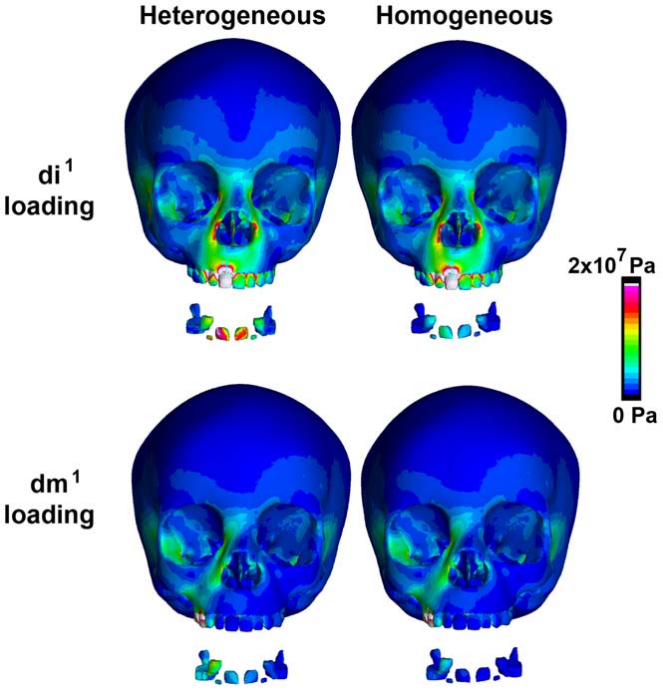
Solved models with secondary tooth caps extracted for illustration. Overall patterns of von Mises stress are similar between models within trials, but not between trials, suggesting bite location (e.g. incisor versus molar) has a greater effect on the pattern of von Mises stress than do the material properties of tooth caps.

Maximum and minimum von Mises stress values in the face were only <1% and 3% lower in the heterogeneous model than the homogeneous model when we loaded the deciduous right central incisor (Table 3). These small differences are mirrored by values of von Mises stress at seven landmarks on the surface of the face (Figures 2–3). Both models exhibit very similar patterns of stress on the external face and nearly identical von Mises stress values at each craniofacial landmark (see points 1–7 in Figure 2b). Von Mises stress values are, on average, 100% greater at embedded permanent tooth cap landmarks (points 8–15 in Figure 2b) in the heterogeneous model relative to the homogeneous model. Although the stiff, enamel-covered tooth caps embedded in the maxilla experience high stresses during loading, those stresses have little or no effect on average von Mises stresses on the surface of the face.

**Table 3:** Landmark percent differences.

	% Difference	
Landmark	Incisor Loading	Molar Loading
1	<1%	5.1
2	<1%	<1%
3	1.6	<1%
4	4.0	9.7
5	2.2	1.7
6	2.0	5.1
7	16.2	4.1
8	122.2	127.1
9	133.7	140.0
10	93.4	125.2
11	95.7	126.1
12	118.9	150.3
13	127.3	133.5
14	157.2	161.9
15	157.3	154.8

The percent difference (%) between von Mises stresses for 15 landmarks under deciduous incisor (di^1^) and molar (dm^1^) loading. Values represent the percentage difference in the heterogeneous model relative to the homogeneous model.

The heterogeneous and homogeneous models exhibited similar patterns of stress throughout the craniofacial region under the unilateral molar load (Figure 3). The highest stress values were localized at landmark 6 on the right maxilla, just superior to the site of load application (likely an artifact of the proximity of that point to the applied load, see Figure 2c). Average von Mises stress values in the embedded permanent tooth caps were greater in the heterogeneous model than to the homogeneous model, although von Mises stresses at the craniofacial landmarks were essentially unchanged (Figure 2c).

## Discussion

We investigated the interaction between material properties and facial geometry in children, and predicted that the presence of unerupted secondary teeth in the maxilla would affect the magnitude and/or distribution of stress in the face during loading. Based on a heuristic model, we predicted that variation in material properties of the tissues within tooth crypts would reduce stress in the face. We found that assigning stiff material properties to the developing permanent tooth caps resulted in much higher von Mises stresses in the developing tooth caps when compared to the model in which the tooth caps were modeled as bone. However, the craniofacial skeleton was largely unresponsive to changes in the material properties of the tooth caps. The hypothesis that the presence of unerupted secondary teeth in the maxilla strengthens the facial skeleton by reducing facial stress is not supported by these FEA results. These results do support many previous studies [44], [45] demonstrating that higher stresses are produced by loading the anteriorly-placed incisors than by loading the posterior dentition.

The hypothesis tested here assumes that children eat harder, more solid foods that have the potential to generate higher masticatory stress in the face during the post-weaning period. Bone and cartilage exhibit their highest levels of phenotypic plasticity during early development [46], [47], and the facial skeleton should be more responsive to masticatory loading in childhood than in adulthood. The extent of this plasticity is uncertain, however, since juvenile mandibles may be more strongly influenced by spatial demands associated with tooth size and position than with masticatory stress *per se* [23], [48]. Moreover, Western weanling diets are often heavily prepared, requiring little oral manipulation by the child, and even hunter-gatherer children's diets are known to be higher in soft, ripe fruits and honey than are adult diets [49], [50]. Although the fracture mechanics of weanling diets are essentially unknown, weanling foods are likely to require more force to process than the diets of like-aged juvenile apes, who subsist almost exclusively on breast milk. More data on the responsiveness of the facial skeleton to loading during different stages of growth and on the material properties of foods being consumed by juvenile anthropoid primates are needed to inform this debate.

It is optimal to validate FE models with *in vivo* experimental data to demonstrate that FE analyses produce realistic patterns of strain [43], [51], [52]. Although we were not able to validate our model with in vivo strain data from human children, the broad topographic distribution of stress in our FE model is consistent with that seen in many other studies [17], [24], [26], [44], [45], [53], [54]. A recent study on the juvenile macaque mandible [23] found generally high congruence between FE models with and without secondary tooth crypts modeled entirely as air, but found a notable increase in mandibular symphyseal strain when tooth crypts were present. Our findings, when considered alongside those results, indicate that an important next step for modeling this complex biological system should include the multiple material properties of other tissues present in the tooth crypt and alveolus.

## Acknowledgments

We are sincerely grateful to Joan Richtsmeier for providing CT scans from the Bosma Collection, and to Daniel Lieberman, Janet Monge, and Tom Schoenemann for providing scan data during the course of this project. Sam Cobb, Frank Marlowe, Casey Holliday, Dave Strait, Paul Constantino, and Anne Delvaux provided helpful discussions, and we also thank Dennis O'Rourke and two anonymous reviewers for their valuable feedback on this manuscript. We also thank Ian Grosse, Sean Werle, Douglas Broadfield, and Robert Blanks for their contributions to the early development of this project.
